# Supplementary material for: Genetic variation in cis-regulatory domains suggests cell type-specific regulatory mechanisms in immunity
Source: Commun Biol. 2023 Mar 28;6:335. doi: 10.1038/s42003-023-04688-3 (PMC10050075; doi:10.1038/s42003-023-04688-3)

Supplementary Table 1a: CRDs discovered for similar (94 samples) or maximum sample size

Type of CRD	Cell Type	Sample Size	CRDs discovered	Sample Size	CRDs discovered
hCRD	Neutrophils	94	6,831	165	7,666
	Monocytes	94	7,660	160	9,287
	T-cells	94	5,480	94	5,480
	LCLs	94	10,497	317	12,583
mCRD	Neutrophils	94	3,828	197	6,112
	Monocytes	94	3,877	196	6,053
	T-cells	94	4,275	132	5,701

Supplementary Table 1b: CRD and CRD associations (CRD-QTLs, CRD-genes, CRD-CRD) discovered in Monocytes, Neutrophils, T-cells and LCLs.

Type of CRD	Type of QTL	Description	Range	Number of CRDs tested in these associations			Number of discoveries at 5% FDR		
				Monocytes	Neutrophils	T-cells	Monocytes	Neutrophils	T-cells
hCRD	CRD-QTL	Genetic variant associated with CRD activity (5% FDR)	Cis	9,287 CRDs	7,666 CRDs	5,480 CRDs	7,050	4,854	1,616
	CRD-gene	Gene associated with CRD activity (5% FDR)	Cis				6,755	6,300	2,239
	CRD-CRD	CRD-CRD associations (1% FDR)	Trans				84,690	159,422	116,658
	TRH	cluster of trans CRD-CRD associations	Trans				308	107	31
mCRD	CRD-QTL	Genetic variant associated with CRD activity (5% FDR)	Cis	6,053 CRDs	6,112 CRDs	5,701 CRDs	4,325	4,363	3,786
	CRD-gene	Gene associated with CRD activity (5% FDR)	Cis				2,300	2,027	1,858
	CRD-CRD	CRD-CRD associations (1% FDR)	Trans				11,719	12,525	14,022
	TRH	cluster of trans CRD-CRD associations	Trans				230	234	262

Supplementary Table 2: Cis co-expressed gene pairs (i.e. genes whose expression is correlated among individuals) (FDR 1%) respectively for neutrophils, monocytes and T-cells.

	Co-Expressed Gene Pairs	
Cell Type	Tested	FDR<0.01
Neutrophils	4,663,276	29,940
Monocytes	6,663,972	46,146
T-cells	5,872,671	13,737

Supplementary Table 3: Table summarizing the enrichment in TFBS for significant CRD-QTLs, with odd ratio>2 and p-value <0.05.

data_type	cell	TFBS	pval	oddsratio
hist	mono	SPI1	8,84E-90	3,48
hist	neut	SPI1	3,11E-47	3,08
hist	tcell	TCF4	1,49E-07	3,04
methyl	mono	ETS2	4,12E-02	3,00
hist	tcell	SPI1	1,25E-15	2,93
hist	mono	STAT1	1,48E-61	2,82
hist	mono	TCF4	1,91E-21	2,77
methyl	neut	SPI1	2,19E-29	2,75
methyl	tcell	TCF4	8,57E-11	2,61
methyl	mono	SPI1	1,01E-24	2,52
methyl	mono	TCF4	1,80E-11	2,49
methyl	neut	TCF4	1,25E-10	2,46
hist	tcell	BCL11A	2,16E-07	2,43
hist	neut	STAT1	7,40E-30	2,43
methyl	mono	MED12	7,73E-07	2,40
hist	mono	BCL11A	6,57E-24	2,39
methyl	tcell	SPI1	3,14E-18	2,38
methyl	neut	BCL11A	1,04E-15	2,34
hist	mono	STAT3	8,71E-56	2,33
hist	tcell	STAT1	1,11E-09	2,29
methyl	mono	PU.1	3,06E-03	2,26
methyl	tcell	BCL11A	7,96E-13	2,26
methyl	mono	BCL11A	1,62E-14	2,23
hist	neut	TCF4	1,26E-08	2,19
methyl	neut	GABP	1,24E-18	2,16
methyl	neut	STAT1	1,50E-19	2,14
methyl	neut	SOX4	4,44E-06	2,13
methyl	mono	SOX4	4,22E-06	2,11
hist	tcell	GABP	2,02E-06	2,10
methyl	tcell	MED12	3,01E-04	2,09
hist	neut	BCL11A	1,79E-11	2,06
methyl	tcell	STAT1	3,91E-15	2,06
methyl	mono	GABP	3,71E-16	2,03
hist	neut	STAT3	2,47E-25	2,01
hist	mono	GABP	1,10E-18	2,00

Supplementary Table 4: Top 20 Gene Ontology terms linked to immune response, from 2 TRHs: one in neutrophils and one in T-cells.

Cell	GOTerm	description	Enrichment	Qvalue
T-cells	GO:0045959	negative regulation of complement activation, classical pathway	30.9	0.00723
T-cells	GO:0030450	regulation of complement activation, classical pathway	30.9	0.00734
T-cells	GO:0045619	regulation of lymphocyte differentiation	3.98	0.000738
T-cells	GO:0002822	regulation of adaptive immune response based on somatic recombination of immune receptors built from immunoglobulin superfamily domains	3.83	0.00745
T-cells	GO:0002819	regulation of adaptive immune response	3.64	0.00691
T-cells	GO:0002250	adaptive immune response	3.49	0.00929
T-cells	GO:0030098	lymphocyte differentiation	3.41	0.00745
T-cells	GO:1902105	regulation of leukocyte differentiation	3.01	0.005
T-cells	GO:0002521	leukocyte differentiation	2.9	0.00963
T-cells	GO:0045087	innate immune response	2.7	0.00256
T-cells	GO:0002683	negative regulation of immune system process	2.62	0.00266
T-cells	GO:0050778	positive regulation of immune response	2.6	5.11e-05
T-cells	GO:0002684	positive regulation of immune system process	2.55	3.89e-07
T-cells	GO:0050776	regulation of immune response	2.53	5.55e-07
T-cells	GO:0002253	activation of immune response	2.5	0.00477
T-cells	GO:0006955	immune response	2.42	9.82e-05
T-cells	GO:0002682	regulation of immune system process	2.34	7.79e-09
Neutrophils	GO:0002682	regulation of immune system process	1.43	0.0026
Neutrophils	GO:0002376	immune system process	1.35	0.00314

Supplementary Table 5a: Significant trans-eQTL (FDR 5%) found for each cell-type.

	Trans eQTL mapping			
	Scenario 1		Scenario 2	
Cell Type	Tested	FDR<0.05	Tested	FDR<0.05
Neutrophils	265,055	55	305,359	62
Monocytes	176,392	16	164,610	65
T-cells	45,767	5	53,420	4

Supplementary Table 5b: Overlap between trans eGenes (aCRD and eGene) across the three immune cell types, with eQTLGen trans-eQTLs.

	Overlap with eQTLGen trans-eQTLs (L'>0.9)	
	Unique hits FDR<0.05	Overlap eQTLGen associations
Neutrophils	117	44
Monocytes	81	1
T-cells	9	1

Supplementary Figure 1: hCRDs across neutrophils, monocytes, T-cells for 3 cell types, for chromosomes 1 to 12

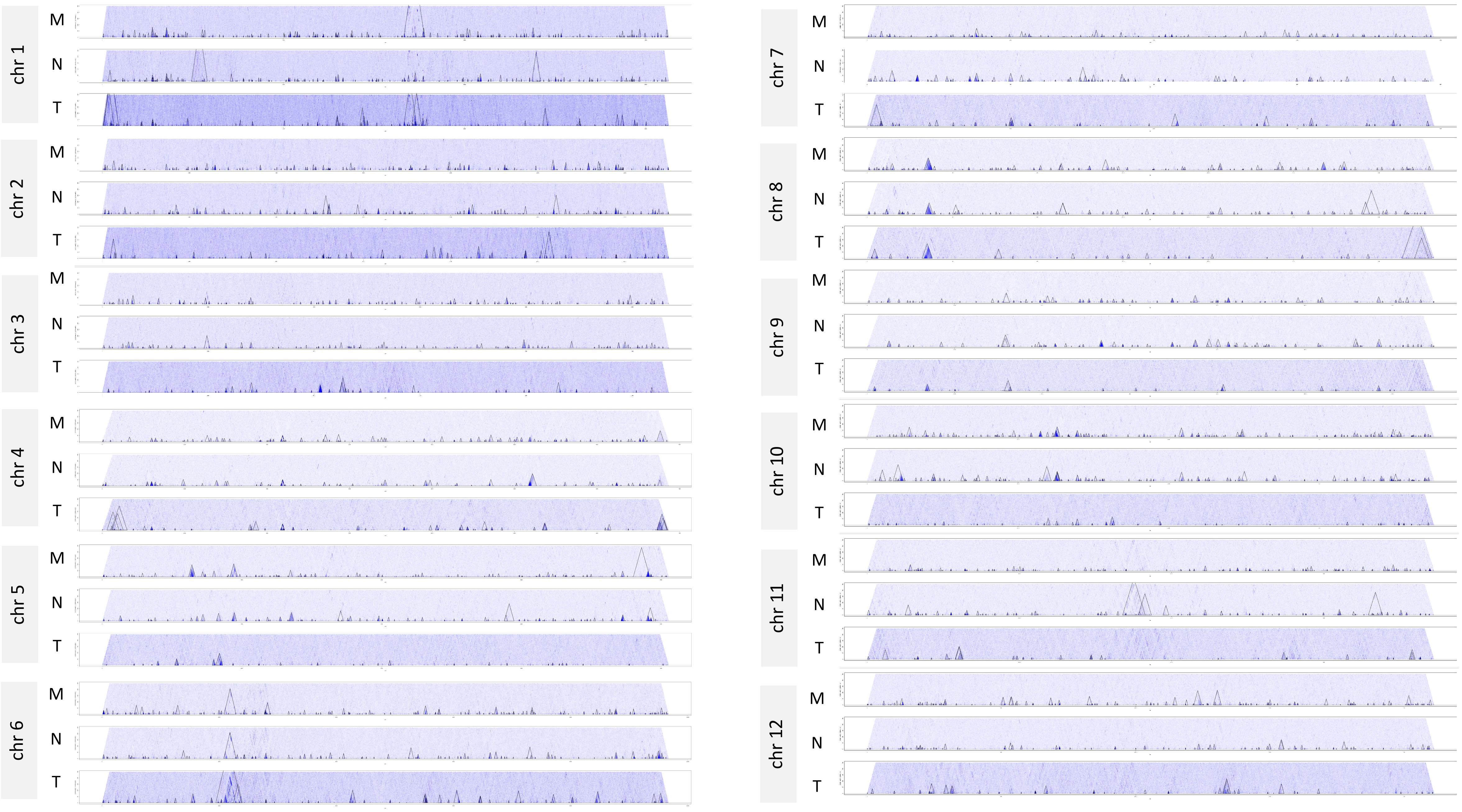

Supplementary Figure 2: hCRDs across neutrophils, monocytes, T-cells for 3 cell types, for chromosomes 13 to 22

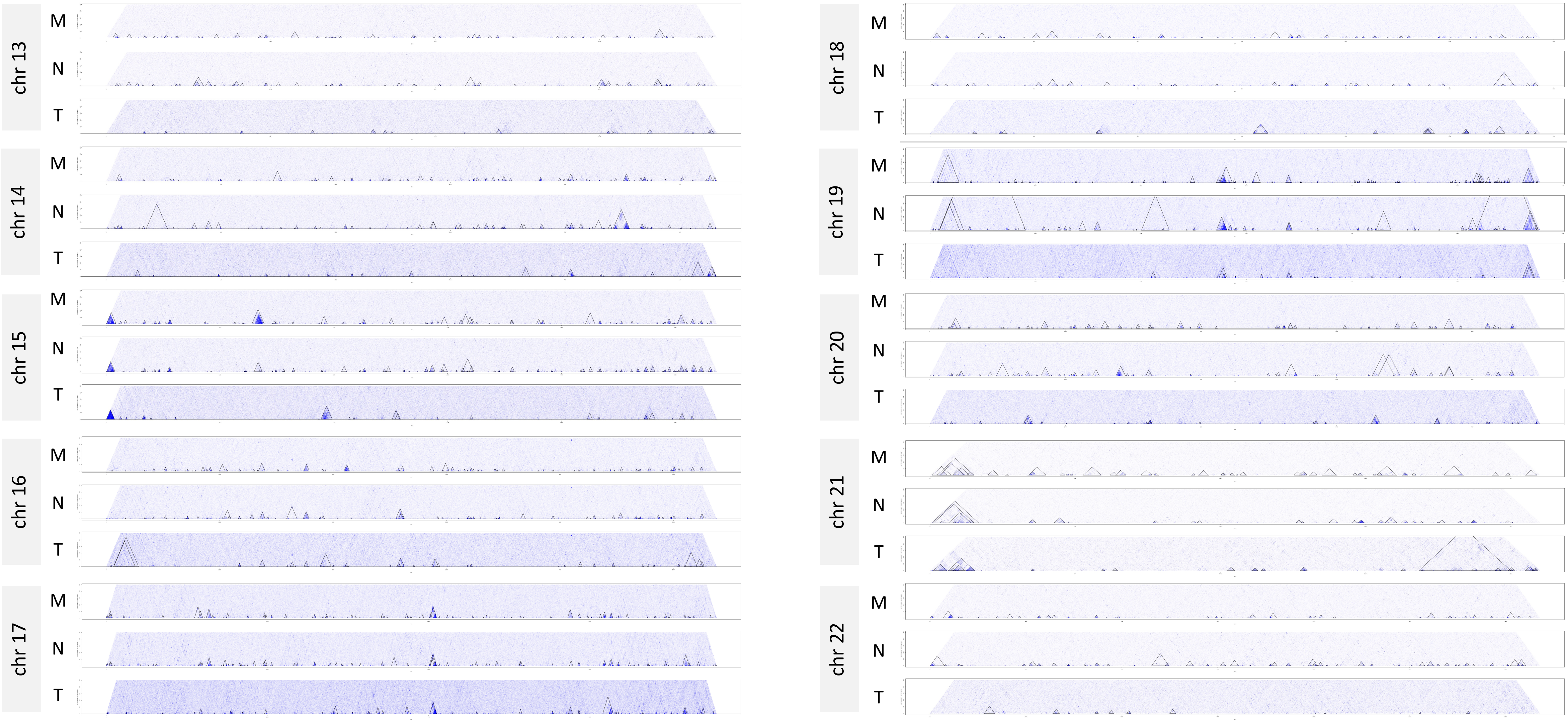

Supplementary Figure 3: Size distribution of mCRDs (top) and mCRDs (bottom) in Neutrophils, Monocytes and T-cells. The median is represented in red and the modelling of mCRD distribution with a mixture gaussian model of small domains (0.2 to a few kb) and large domains (a few kb to 1-2Mb) is displayed in red and green.The threshold corresponding to the 0.95 percentile is plotted in black.

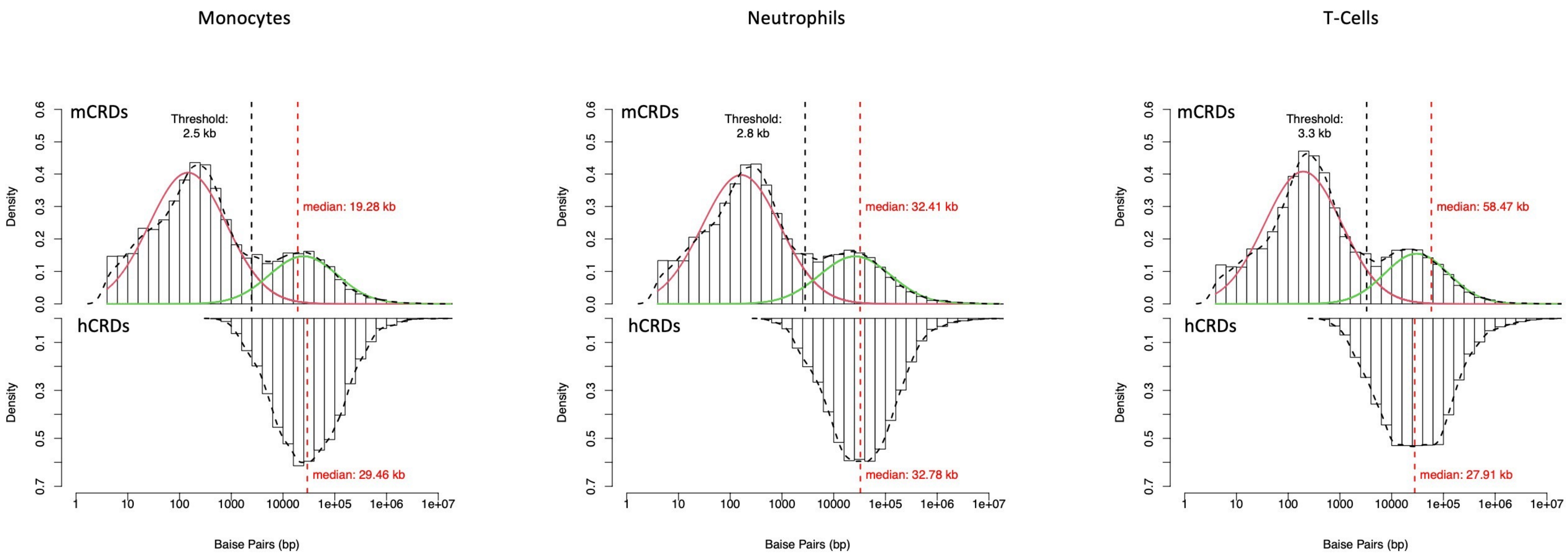

Supplementary Figure 4: Tissue sharing of hCRDs (top) and mCRDs (bottom) across neutrophils, monocytes and T- cells for an identical sample size of 94 samples. A CRD is shared between two cell types more than 50% of the peaks of the reference CRD are present in a CRD from the query tissue. The fraction of sharing is shown for every combination of tissues.

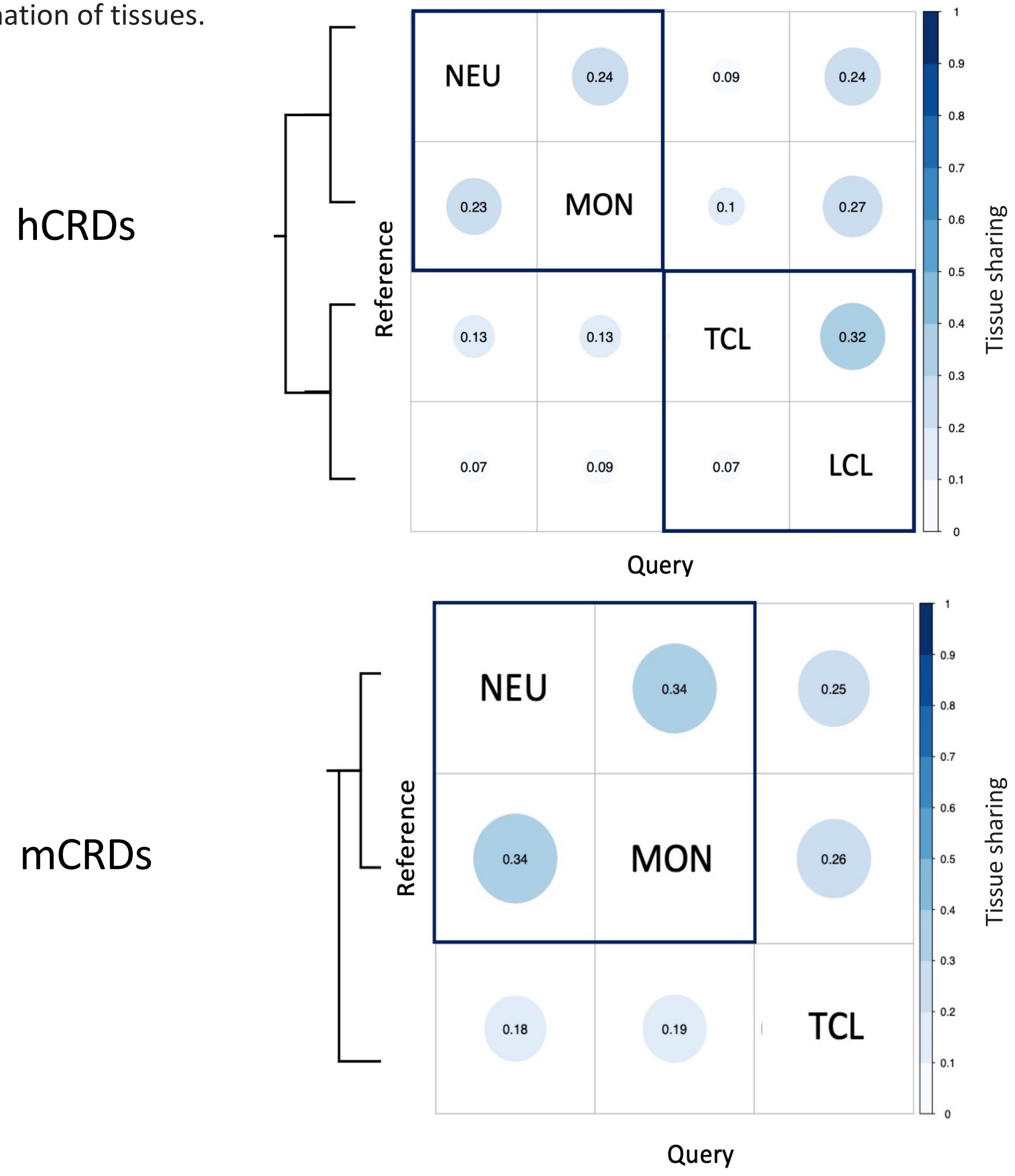

Supplementary Figure 5: Overlap across cell types for chromatin peaks (top) and CpG marks (bottom) belonging to hCRDs.

Chromatin peaks

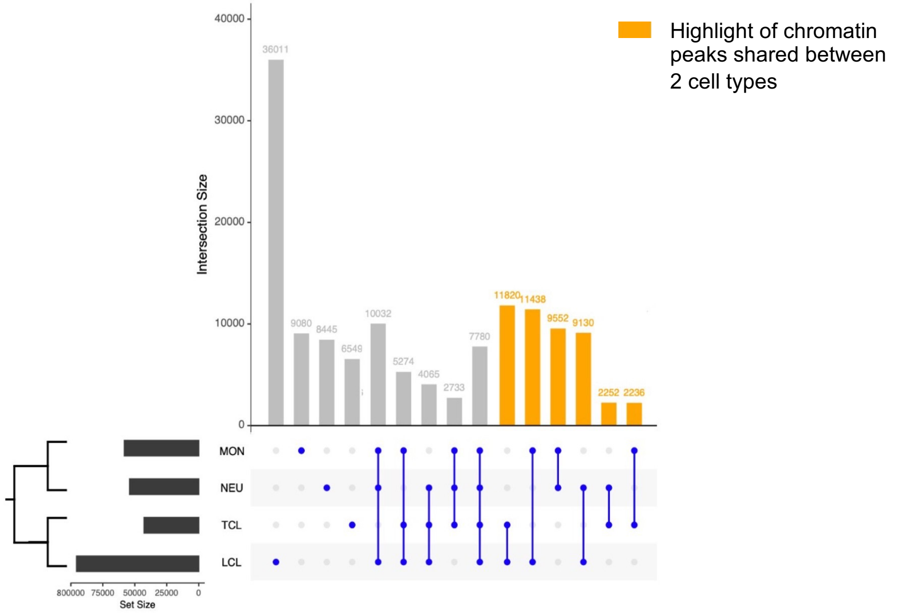

CpG marks

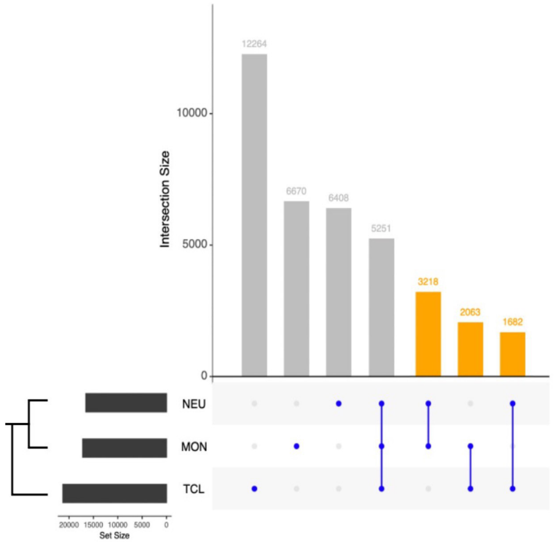

Supplementary Figure 6: hCRD-gene(left) and mCRD-gene(right) association sharing (blue) at 5% FDR. A CRD- gene association is shared between two cell types if the reference CRD and gene are also associated in the query cell type. The fraction of sharing is shown for every combination of tissues. Fraction of genes shared between cell-types (red). We considered the genes involved in the CRD-gene associations (5% FDR)

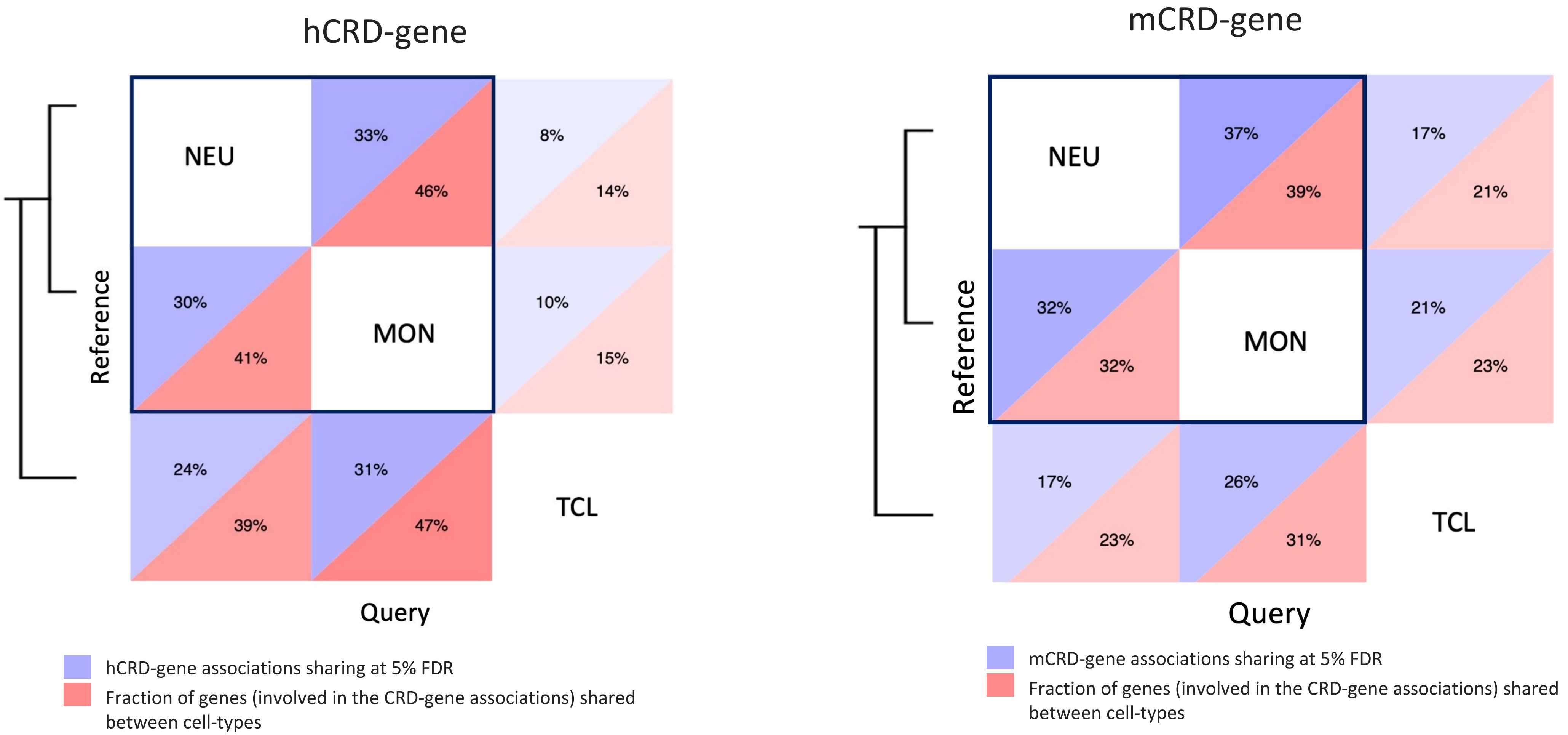

Supplementary Figure 7: Fraction of CRDs or genes as a function of the number of genes or CRDs they are associated with, respectively for hCRDs, mCRDs in neurophils, monocytes and T-cells.

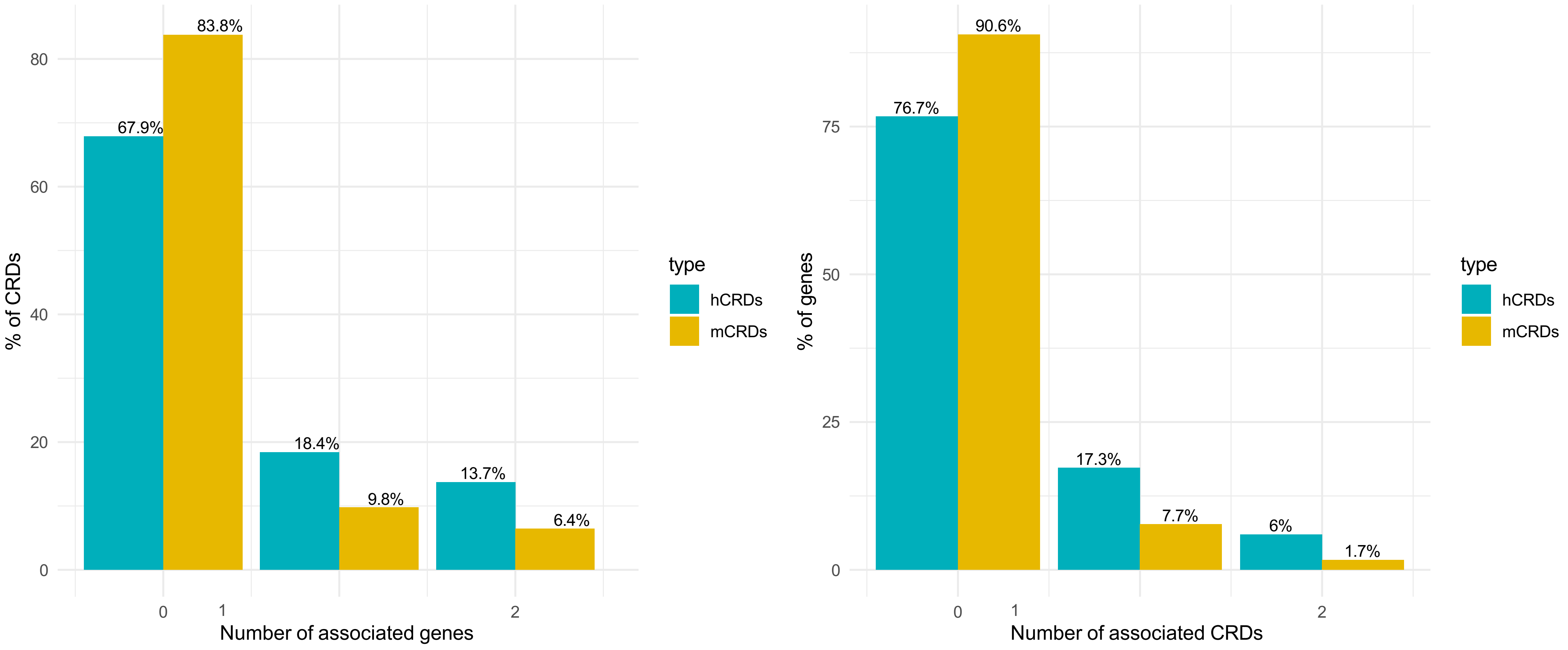

Supplementary Figure 8: Percentages of pairs of co-expressed genes (5%FDR) that associate with the same hCRD(top) / mCRD (bottom) as a function of distance between genes. Gene pairs odds ratios of belonging to the same CRD while being co-expressed are shown in parentheses. Each bar is stratified by the mean distance between pair of genes and their associated CRDs. The position of the genes according to the CRD is color coded on the figure.

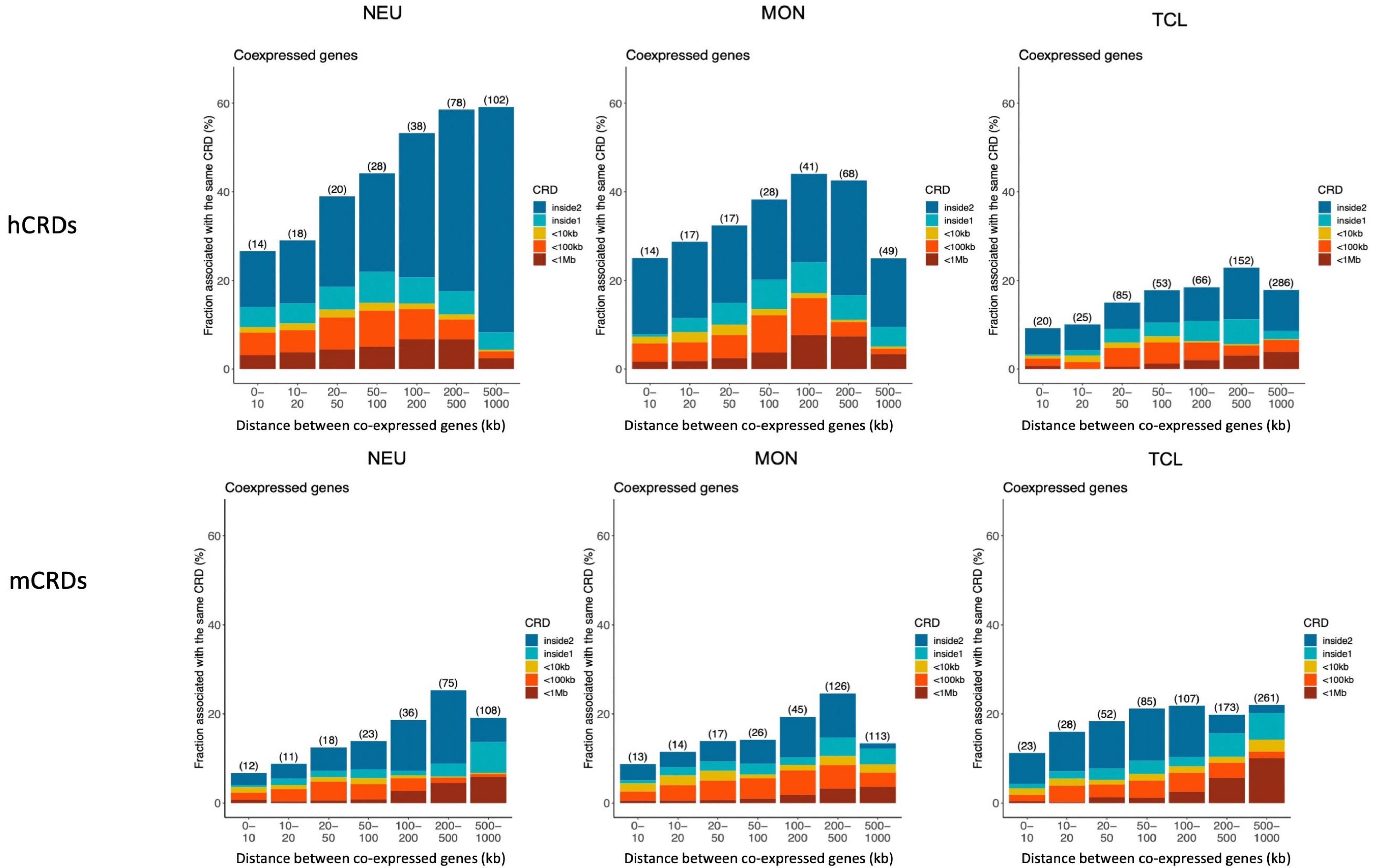

Supplementary Figure 9: $\pi 1$ estimate of hCRD-QTL (blue) and mCRD-QTL (red) sharing between cell-types

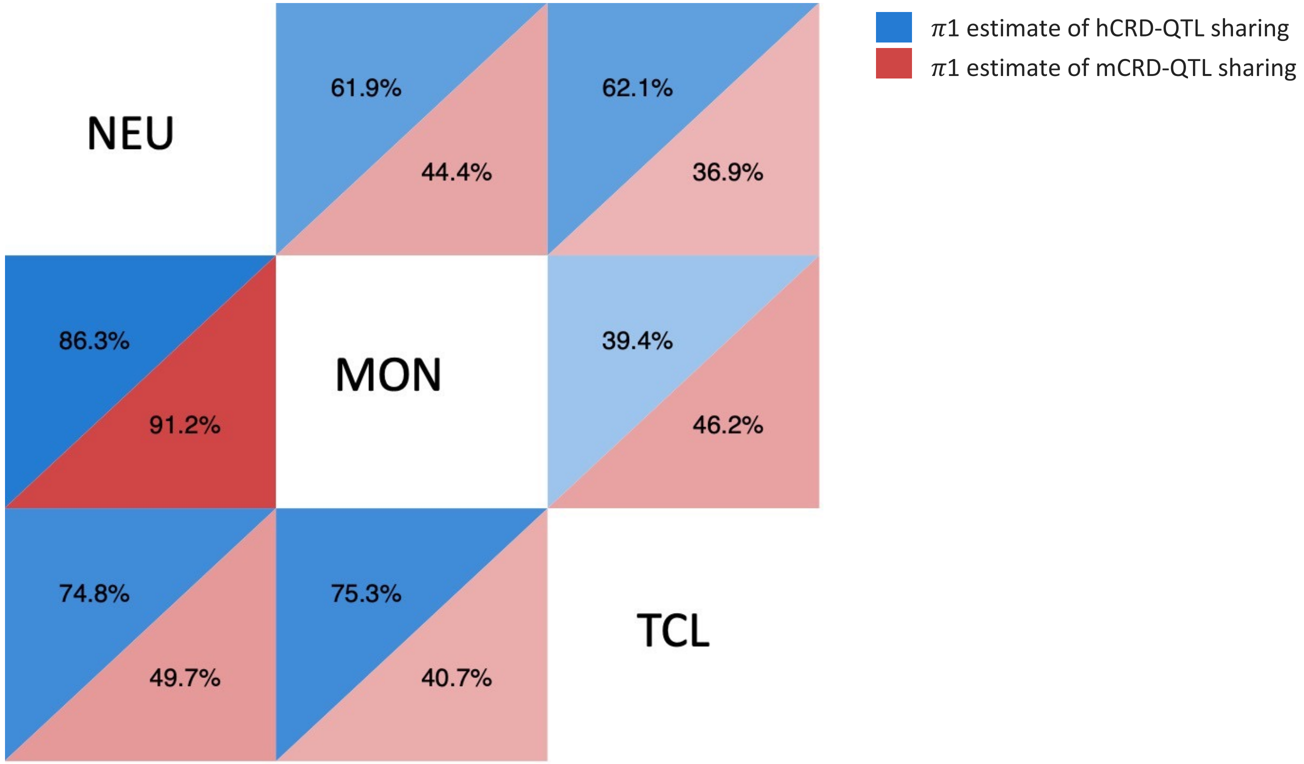

Supplementary Figure 10: Minor allele frequencies (MAF) of histone and methyl CRD-QTLs among the 3 cells studied.

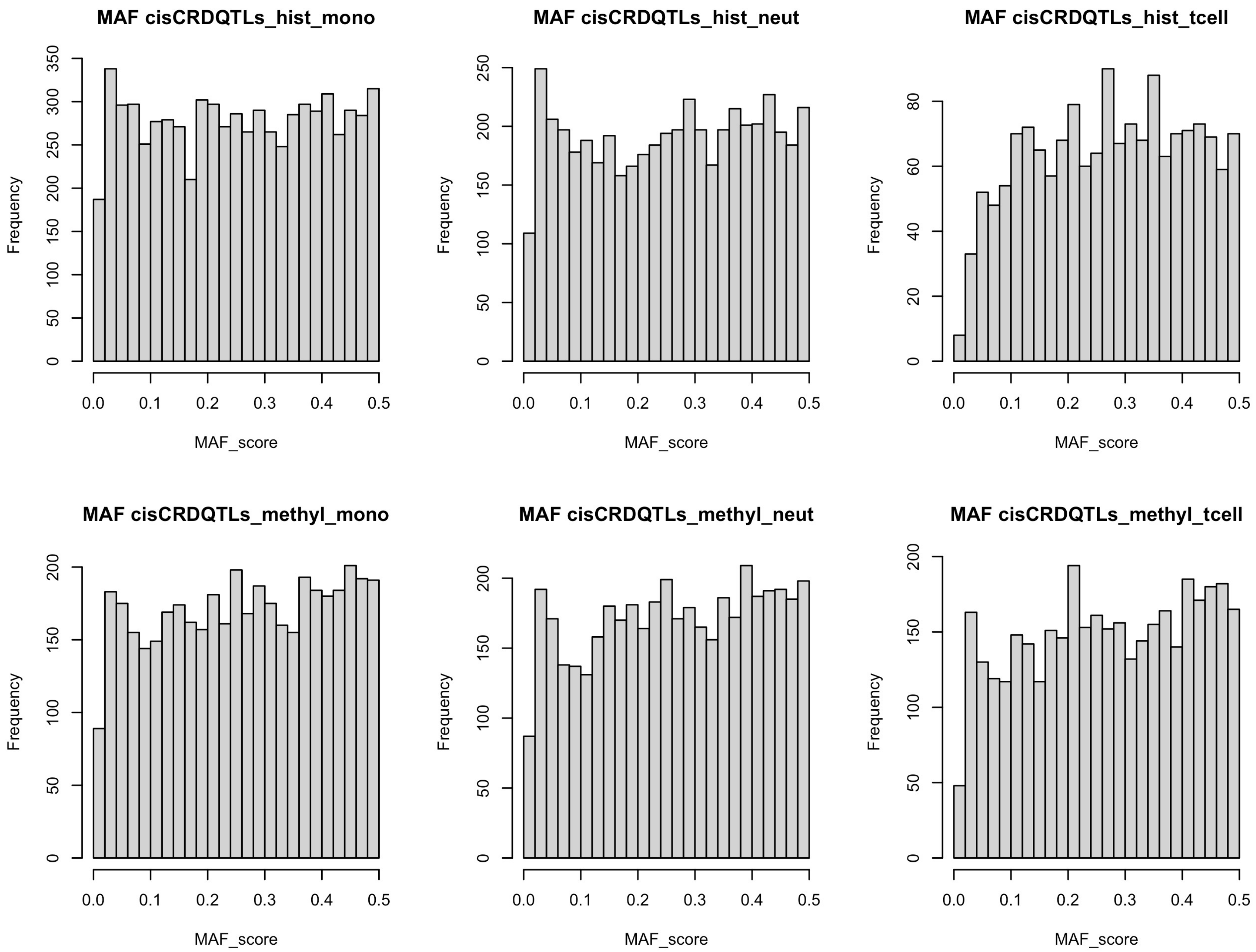

Supplementary Figure 11: Enrichment in the 50 most represented TFs for significant CRD-QTLs. Labels present for TFBS with an odd ratio > 2 and p-value>10^{-4}

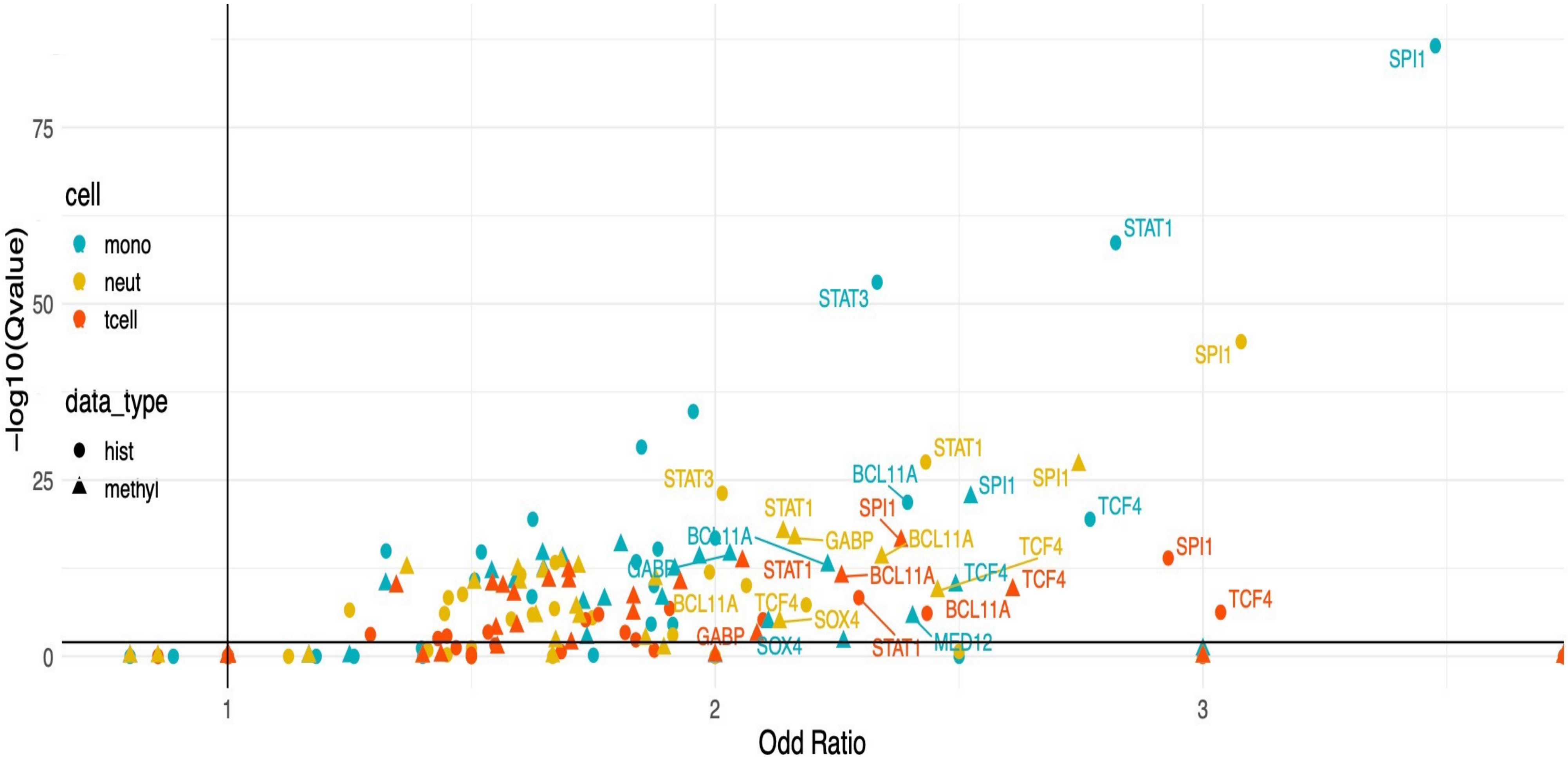

Supplementary Figures 12a,b: Quantile-quantile (Q-Q) plots and genomic inflation factor (λ metric) for hCRD-QTLs (a) and mCRD-QTL (b) for 7 autoimmune diseases (celiac disease [CE], inflammatory bowel disease [IBD], Crohn's disease [CD], ulcerative colitis [UC], multiple sclerosis [MS], Type 1 diabetes [DT1] and rheumatoid arthritis [RA]), Type 2 diabetes [DT2] as control, and 7 blood traits (BT): Basophil count (BC), Eosinophil count (EC), Red blood cell count (RBC), White blood cell count (WBC), Lymphocyte count (LC), Neutrophil count (NC) and Monocyte count (MC).

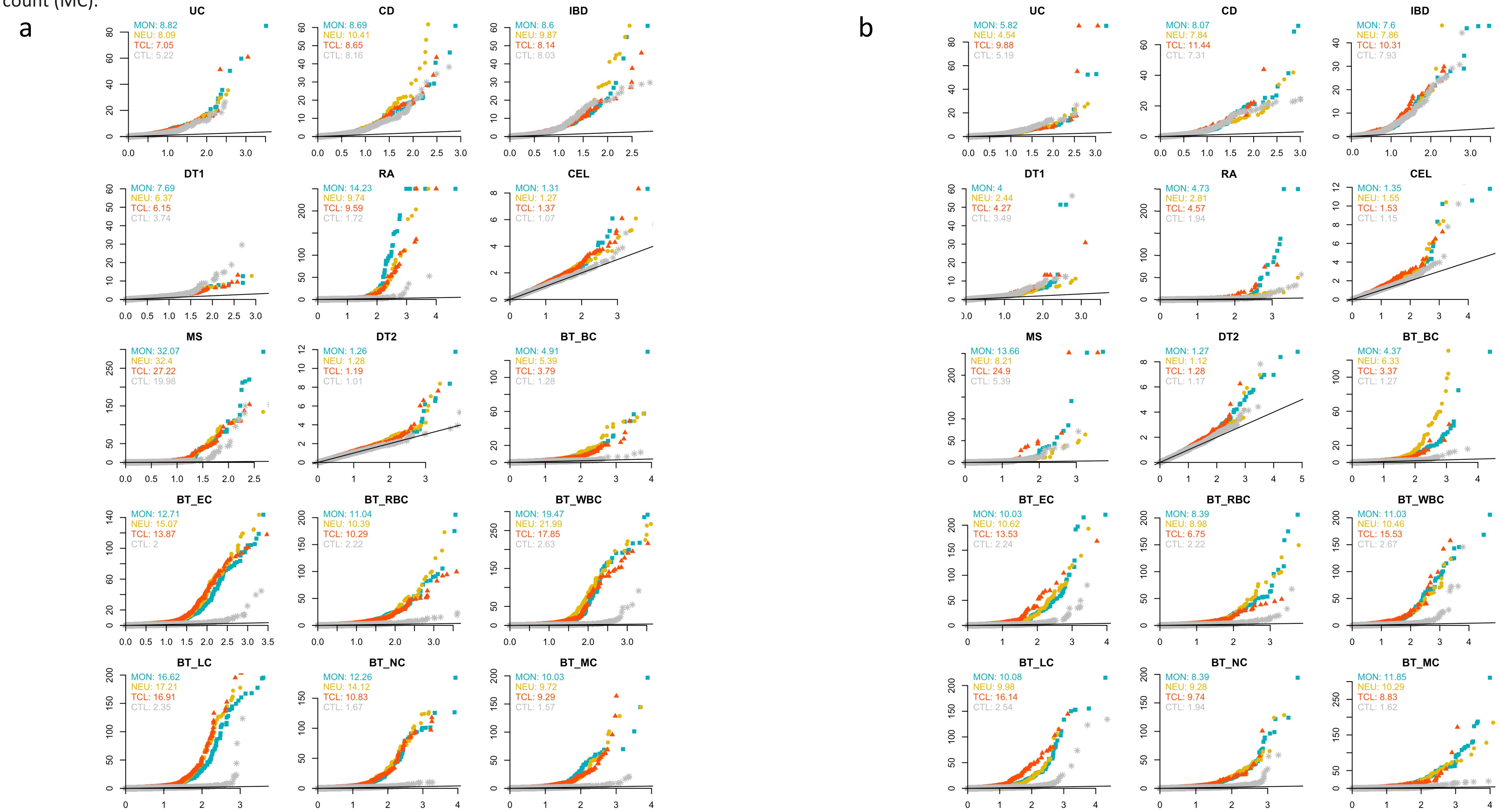

Supplementary Figure 13a: Percentages of significant association at 1% FDR between pairs of peaks located onthe same chromosome as a function of PCHi-C signal (CHiCAGO score).

Supplementary Figure 13b: Fraction of monocytes chromatin peak pairs on the same chromosome supported by PCHi-C data (CHiCAGO score >5) at significantly associated (pink) and non-associated (blue) pairs of chromatin peaks within bins of increasing distance between peaks.

Supplementary Figure 13c:Fraction of T-cells chromatin peak pairs on the same chromosome supported by PCHi- C data (CHiCAGO score >5) at significantly associated (pink) and non-associated (blue) pairs of chromatin peaks within bins of increasing distance between peaks.

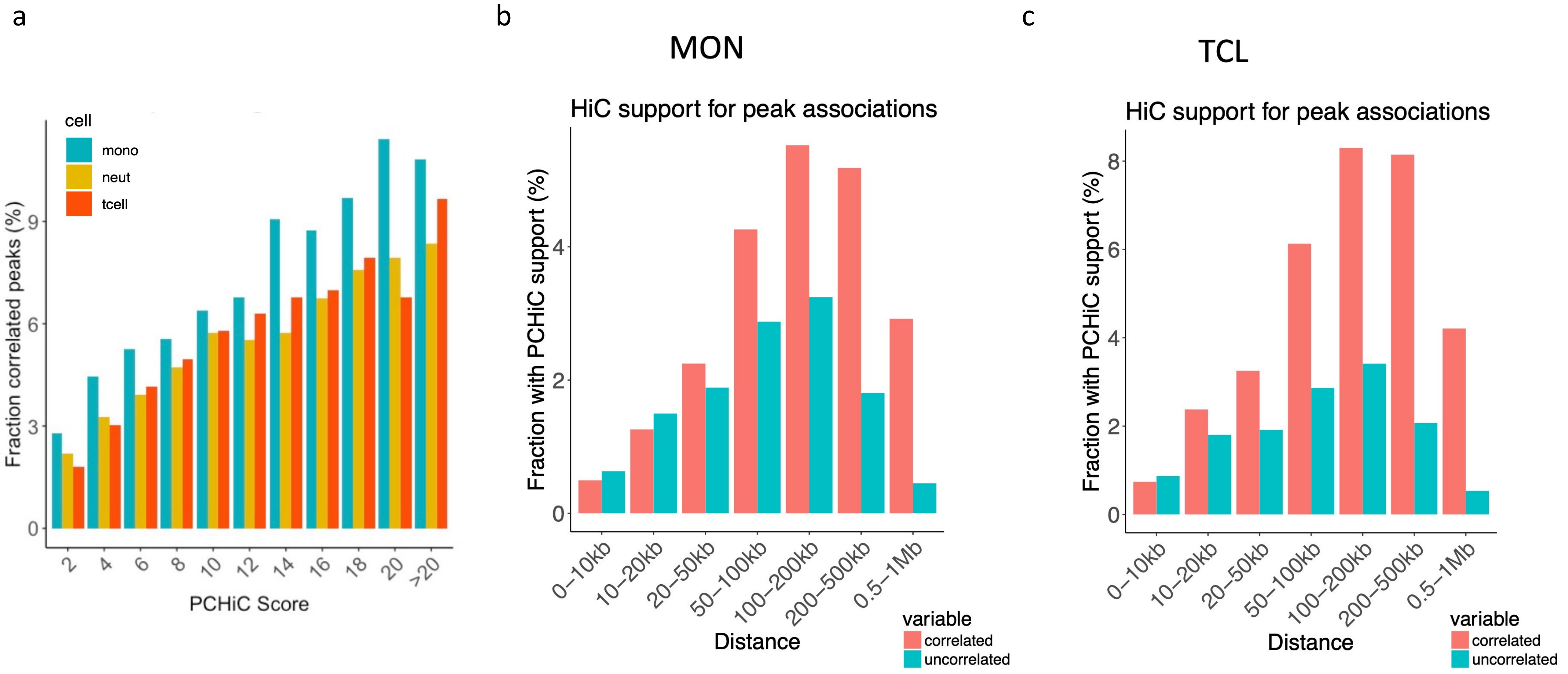

Supplementary Figure 14: Distribution of nominal p-values for CRD-CRD trans associations in neutrophils, monocytes and T cells. The number of significant associations at FDR=1% is given above each histogram. On the bottom, same figures for a similar sample size (n=94) across cell- types.

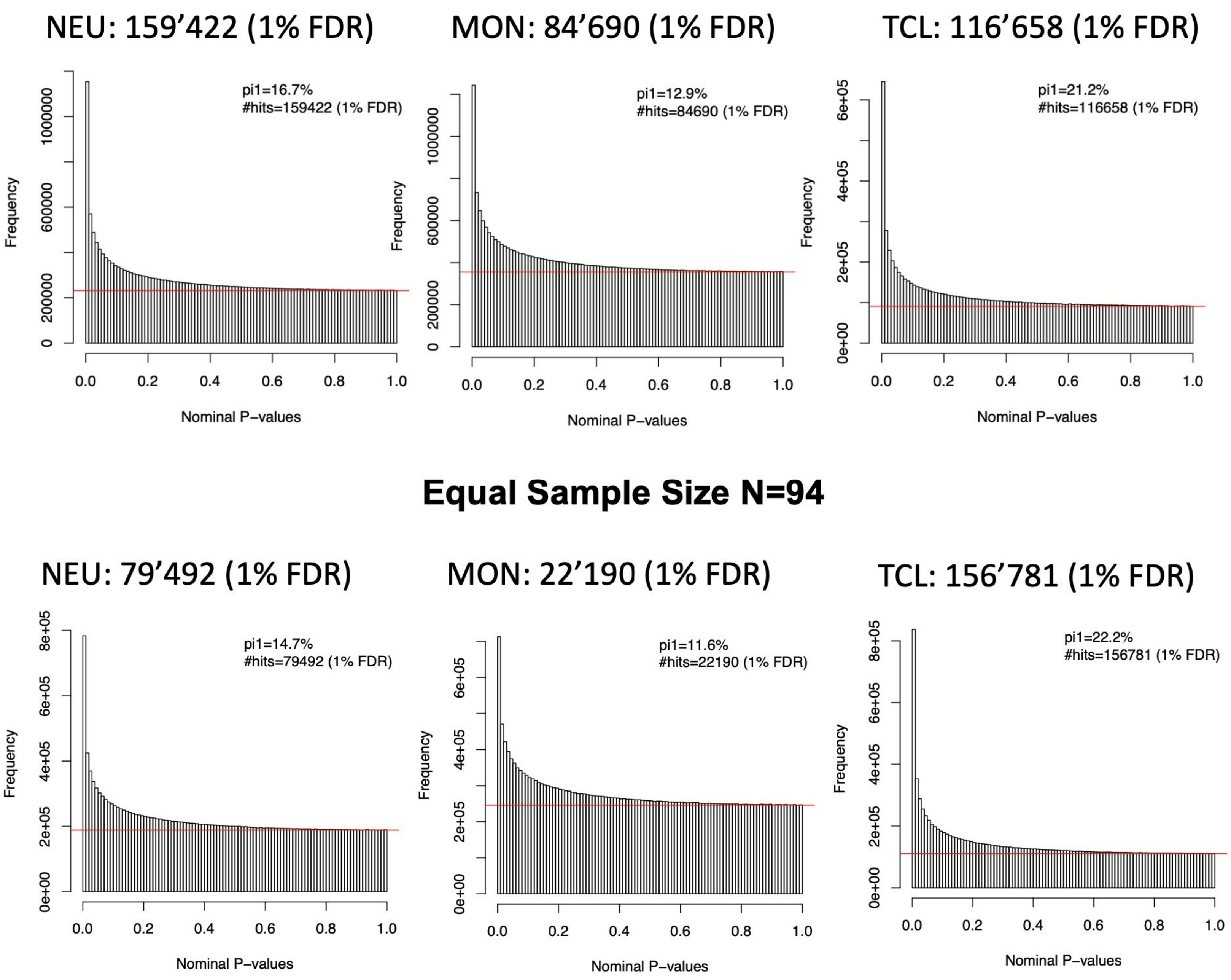

Supplementary Figure 15: Network representation of the 1,000 strongest associations between histone CRDs on distinct chromosomes for neutrophils, monocytes and T cells. Each network node is a CRD and each edge is a significant association between two CRDs. Nodes are colored by chromosome number.

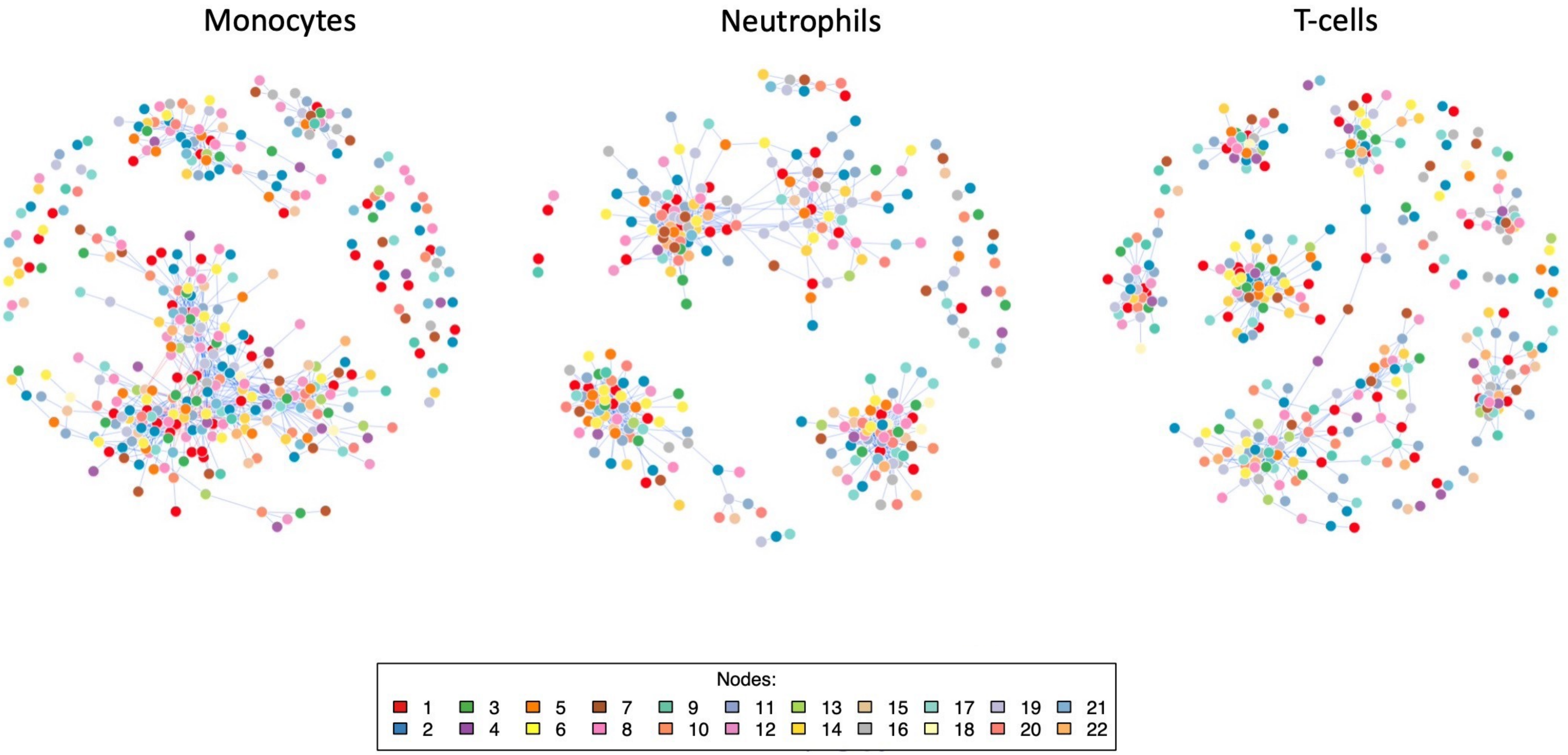

Supplementary Figure 16a: Distribution of histone and methyl TRH sizes for a maximum of 50 TRHs across cell types

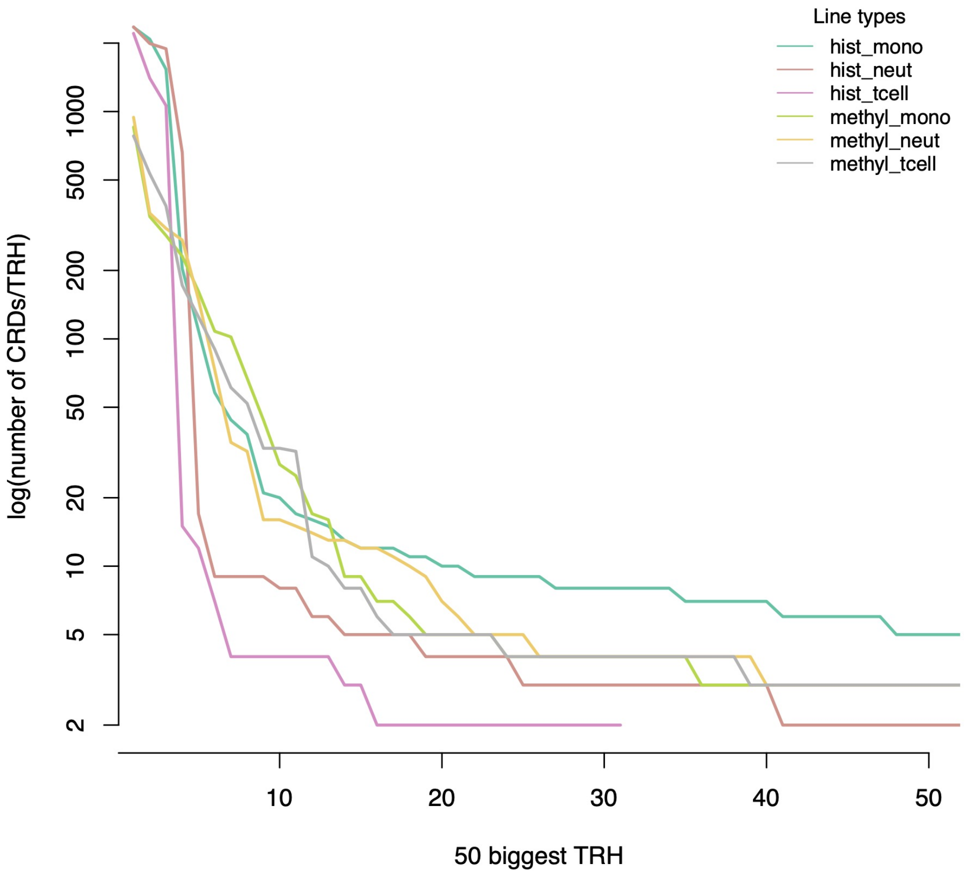

Supplementary Figure 16b: Connectivity of hCRDs in CRD-CRD trans networks across cell types.

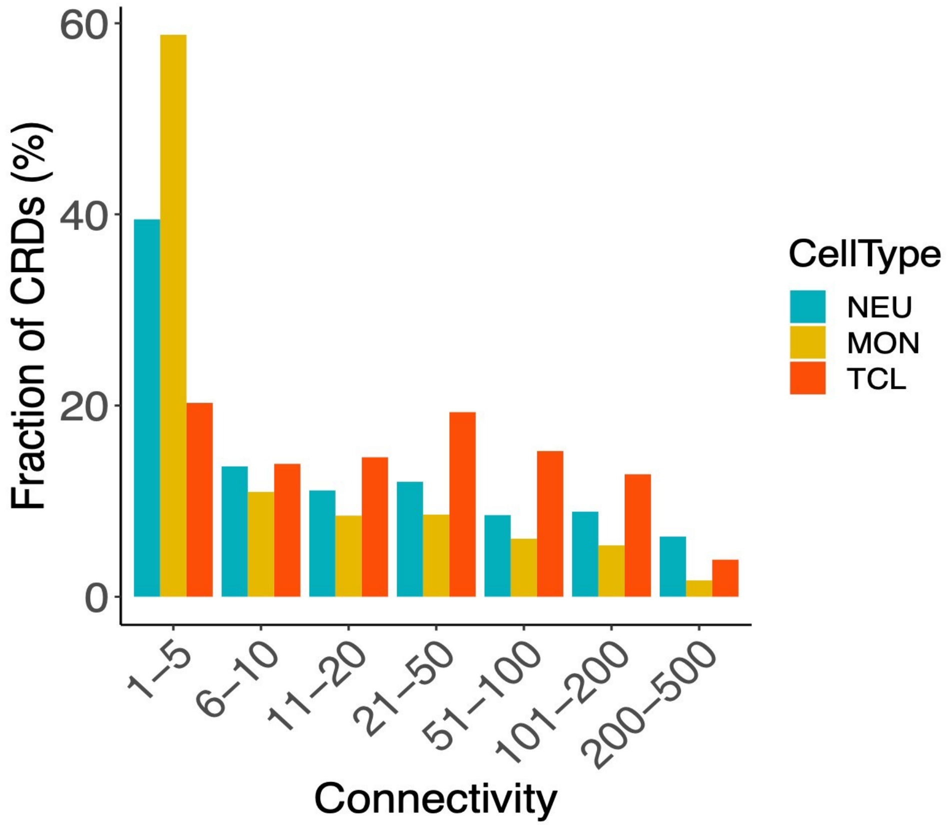

Supplementary Figure 17: Patterns of trans hCRD-hCRD association sharing were also estimated by extracting the significant trans CRD associations identified in one cell-type and then tested for replication in another cell type

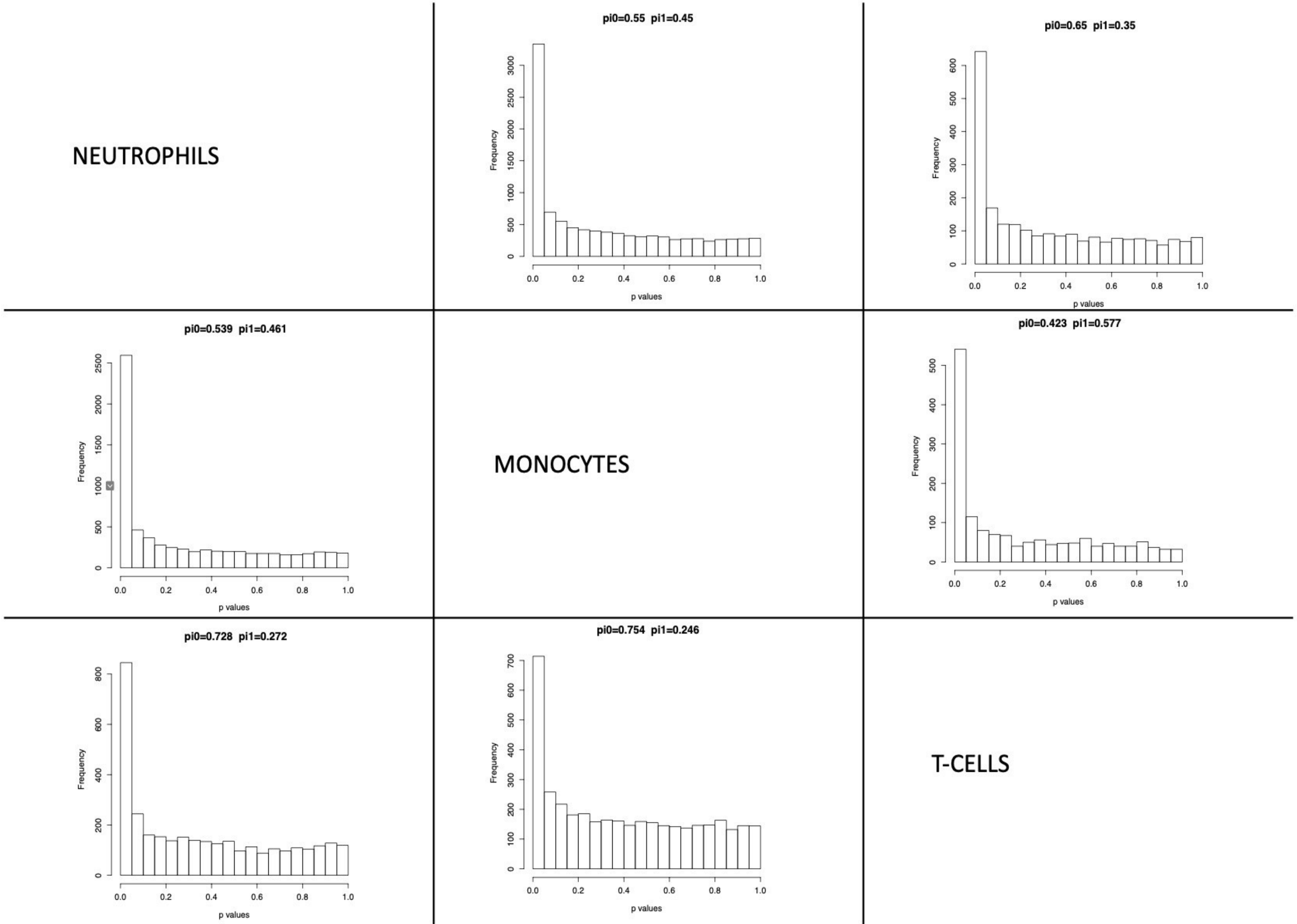

Supplementary Figure 18: Patterns of trans mCRD-mCRD association sharing were also estimated by extracting the significant trans CRD associations identified in one cell-type and then tested for replication in another cell type

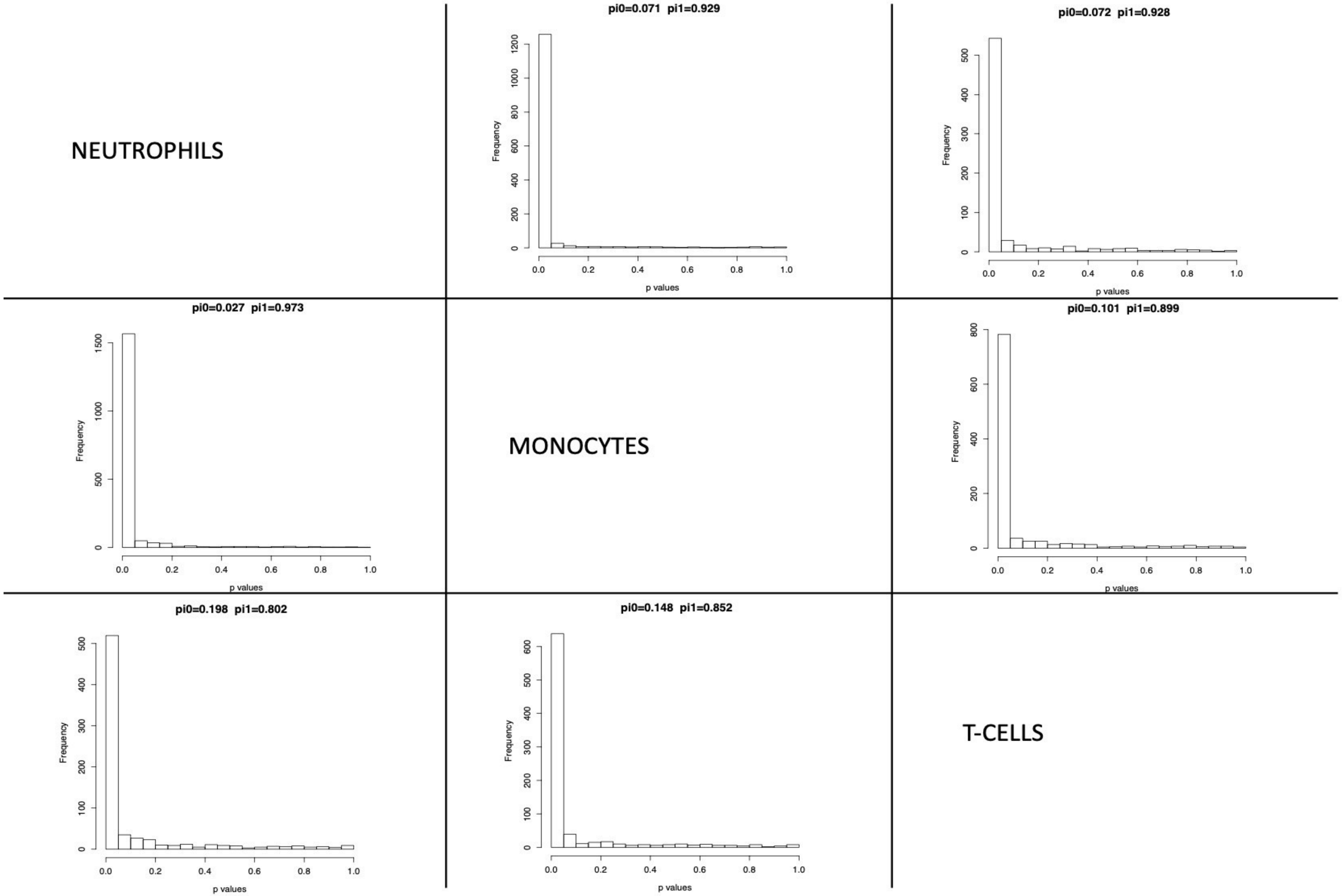

Supplementary Figure 19: Gene Set Enrichment Analysis of genes belonging in a TRH. Enrichment of GO Term as a function of q- value for 3 cell types.

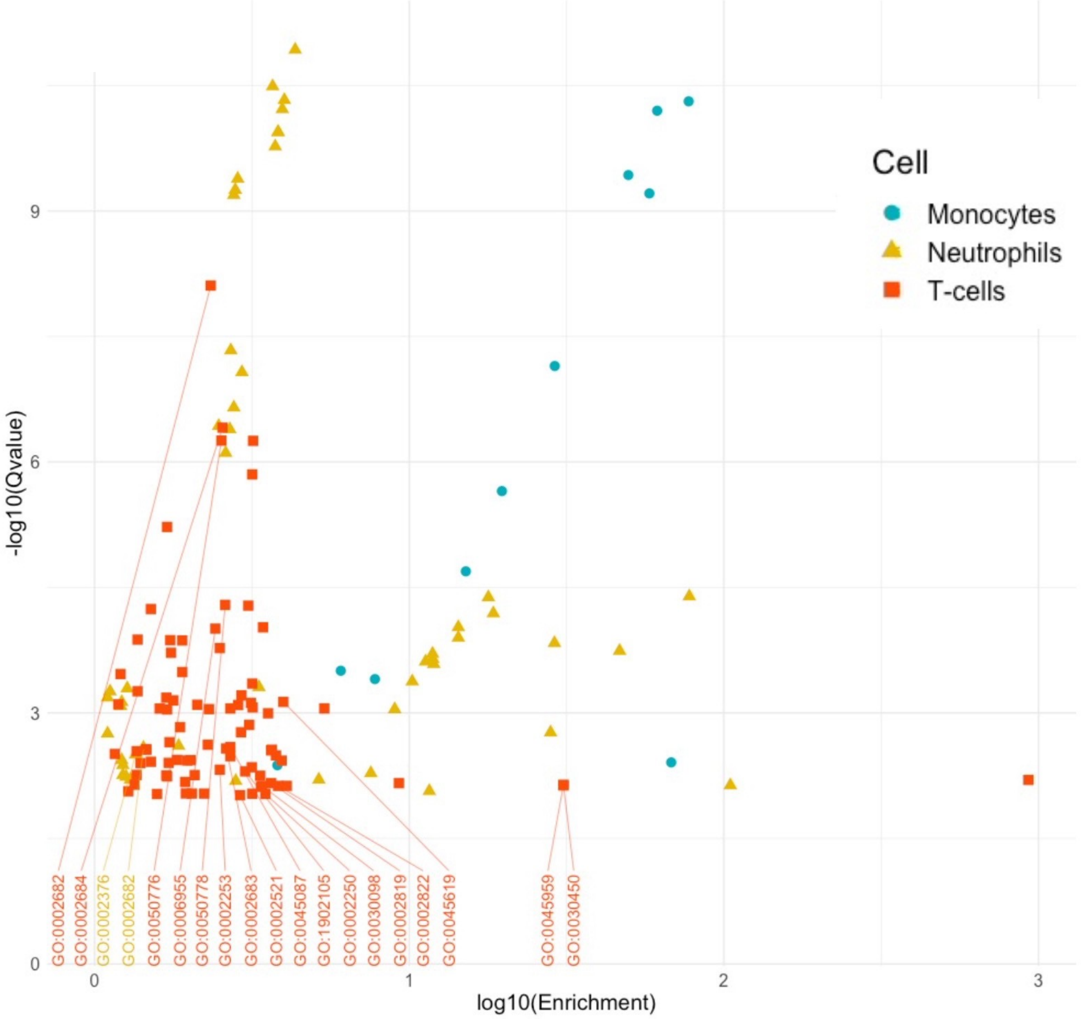

Supplementary Figure 20: Schematic of the different data integration strategies to link variants to genes on different chromosomes and QQplots of trans-eQTL nominal P-values for neutrophils, monocytes and T cells.

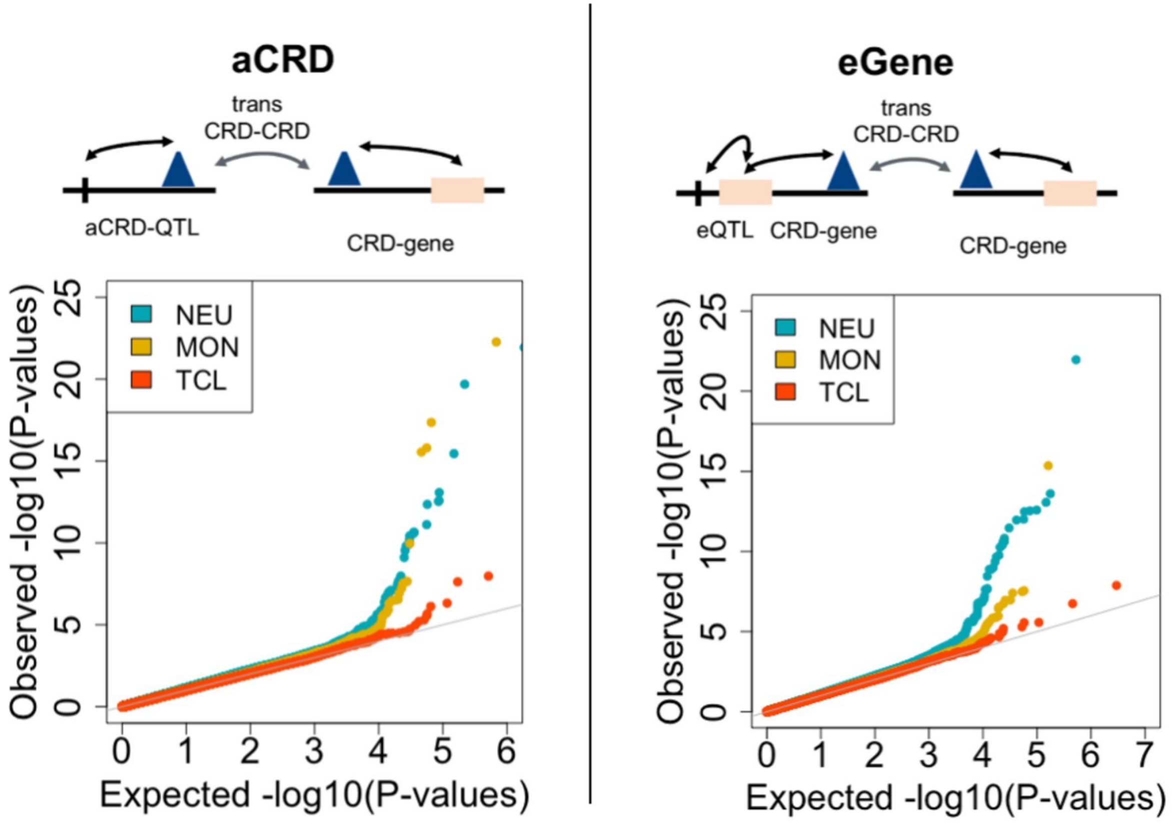

Supplement: Supplementary file 1 — Supplementary Information [file 42003_2023_4688_MOESM1_ESM.pdf]
