# Supplementary material for: Genetic variation in cis-regulatory domains suggests cell type-specific regulatory mechanisms in immunity
Source: Commun Biol. 2023 Mar 28;6:335. doi: 10.1038/s42003-023-04688-3 (PMC10050075; doi:10.1038/s42003-023-04688-3)
Supplement: Supplementary file 2 — Description of Additional Supplementary Files [file 42003_2023_4688_MOESM2_ESM.pdf]

Description of Additional Supplementary Files

File Name: Supplementary Data 1
Description: CRD-gene

File Name: Supplementary Data 2
Description: CRD-QTL

File Name: Supplementary Data 3
Description: Significant trans CRD associations (FDR 1%)

File Name: Supplementary Data 4
Description: Trans-eQTL scenario 1 (aCRD) and scenario 2 (eGenes) at FDR 5%

File Name: Supplementary Data 5
Description: Information about supplementary datasets
