# Supplementary material for: Genetic variation in cis-regulatory domains suggests cell type-specific regulatory mechanisms in immunity
Source: Commun Biol. 2023 Mar 28;6:335. doi: 10.1038/s42003-023-04688-3 (PMC10050075; doi:10.1038/s42003-023-04688-3)
Supplement: Supplementary file 8 — Reporting Summary [file 42003_2023_4688_MOESM8_ESM.pdf]

Corresponding author(s): Olivier Delaneau, Diana Avalos

Last updated by author(s): 22/02/2023

Reporting Summary

Nature Portfolio wishes to improve the reproducibility of the work that we publish. This form provides structure for consistency and transparency in reporting. For further information on Nature Portfolio policies, see our Editorial Policies and the Editorial Policy Checklist.

Statistics

For all statistical analyses, confirm that the following items are present in the figure legend, table legend, main text, or Methods section.

n/a	Confirmed	
☐	☒	The exact sample size (*n*) for each experimental group/condition, given as a discrete number and unit of measurement
☒	☐	A statement on whether measurements were taken from distinct samples or whether the same sample was measured repeatedly
☐	☒	The statistical test(s) used AND whether they are one- or two-sided<br>*Only common tests should be described solely by name; describe more complex techniques in the Methods section.*
☐	☒	A description of all covariates tested
☐	☒	A description of any assumptions or corrections, such as tests of normality and adjustment for multiple comparisons
☐	☒	A full description of the statistical parameters including central tendency (e.g. means) or other basic estimates (e.g. regression coefficient) AND variation (e.g. standard deviation) or associated estimates of uncertainty (e.g. confidence intervals)
☐	☒	For null hypothesis testing, the test statistic (e.g. *F*, *t*, *r*) with confidence intervals, effect sizes, degrees of freedom and *P* value noted<br>*Give P values as exact values whenever suitable.*
☐	☒	For Bayesian analysis, information on the choice of priors and Markov chain Monte Carlo settings
☒	☐	For hierarchical and complex designs, identification of the appropriate level for tests and full reporting of outcomes
☐	☒	Estimates of effect sizes (e.g. Cohen's *d*, Pearson's *r*), indicating how they were calculated

Our web collection on statistics for biologists contains articles on many of the points above.

Software and code

Policy information about availability of computer code

Data collection	No software was used for the data collection of this study.<br>Data was downloaded from the European Genome-Phenome Archive and from Javierre et al., Cell 2016
Data analysis	Publicly available code used in this study :<br>BCFtools v1.8 (based on HTSlib v1.8)<br>PLINK v1.90b5<br>R v3.5.1<br>HOMER v4.9 (webpage: http://homer.ucsd.edu/homer/ngs/peaks.html)<br>Clomics v1.0 (https://github.com/odelaneau/clomics)<br>QTLTools v1.3.1 (https://qtltools.github.io/qtltools)<br>GOrilla for identifying and visualizing enriched GO terms (http://cbl-gorilla.cs.technion.ac.il/)<br><br>code in open access: https://github.com/dianamatata/CRD_immune_cells and archived at zenodo (https://zenodo.org/record/7660407)

For manuscripts utilizing custom algorithms or software that are central to the research but not yet described in published literature, software must be made available to editors and reviewers. We strongly encourage code deposition in a community repository (e.g. GitHub). See the Nature Portfolio guidelines for submitting code & software for further information.

Data

Policy information about availability of data

All manuscripts must include a data availability statement. This statement should provide the following information, where applicable:

- Accession codes, unique identifiers, or web links for publicly available datasets
- A description of any restrictions on data availability
- For clinical datasets or third party data, please ensure that the statement adheres to our policy

Data downloaded from the European Genome-Phenome Archive
website: https://ega-archive.org/datasets
datasets used:
EGAD00001002663 Illumina HiSeq 2000, 193 samples
EGAD00010000850 DNA methylation profiles of monocytes, neutrophils and T cells from 525 healthy donors
EGAD00001002675 RNA-Seq data for 205 mature neutrophil sample(s)
EGAD00001002670 ChIP-Seq data for 182 mature neutrophil sample(s).
EGAD00001002671 RNA-Seq data for 212 CD4-positive, alpha-beta T cell sample(s).
EGAD00001002673 ChIP-Seq data for 154 CD4-positive, alpha-beta T cell sample(s).
EGAD00001002672 ChIP-Seq data for 172 CD14-positive, CD16-negative classical monocyte sample(s).
EGAD00001002674 RNA-Seq data for 197 CD14-positive, CD16-negative classical monocyte sample(s).

and Hi-C data from Javierre et al., Cell 2016

Human research participants

Policy information about studies involving human research participants and Sex and Gender in Research.

Reporting on sex and gender	Sex was collected by the European Genome-Phenome Project. We have a balanced repartition in the sex of participants (54% females) , and this information was used as covariate in our study. We don't generate sex disaggregated analysis.
Population characteristics	As part of the BLUEPRINT epigenome project, the 200 blood donors are ascertained to be free of disease and representative of the United Kingdom (UK) population at large.<br>more details: https://europepmc.org/article/MED/27863251
Recruitment	The participants were recruited as part of the BLUEPRINT epigenome project
Ethics oversight	European Genome-Phenome Archive, BLUEPRINT epigenome project

Note that full information on the approval of the study protocol must also be provided in the manuscript.

Field-specific reporting

Please select the one below that is the best fit for your research. If you are not sure, read the appropriate sections before making your selection.

☒ Life sciences ☐ Behavioural & social sciences ☐ Ecological, evolutionary & environmental sciences

For a reference copy of the document with all sections, see nature.com/documents/nr-reporting-summary-flat.pdf

Life sciences study design

All studies must disclose on these points even when the disclosure is negative.

Sample size	For each cell-type, we used the maximum number of individuals available and we repeated some analyses using a fixed sample size (n=94) to check whether the number of significant associations depended strongly on sample size
Data exclusions	No data were excluded from this analysis.
Replication	All of the softwares used for this study are publicly available.
Randomization	Randomization was not used since there are no experimental groups.
Blinding	Blinding is not relevant to this study since no group allocation occurs.

Reporting for specific materials, systems and methods

We require information from authors about some types of materials, experimental systems and methods used in many studies. Here, indicate whether each material, system or method listed is relevant to your study. If you are not sure if a list item applies to your research, read the appropriate section before selecting a response.

Materials & experimental systems

n/a	Involved in the study
☒	☐ Antibodies
☒	☐ Eukaryotic cell lines
☒	☐ Palaeontology and archaeology
☒	☐ Animals and other organisms
☒	☐ Clinical data
☒	☐ Dual use research of concern

Methods

n/a	Involved in the study
☒	☐ ChIP-seq
☒	☐ Flow cytometry
☒	☐ MRI-based neuroimaging
